# A novel, clinically relevant use of a piglet model to study the effects of anesthetics on the developing brain

**DOI:** 10.1186/s40169-015-0079-9

**Published:** 2016-01-12

**Authors:** Emmett E. Whitaker, Bruno Bissonnette, Andrew D. Miller, Tanner L. Koppert, Joseph D. Tobias, Christopher R. Pierson, Fievos L. Christofi

**Affiliations:** Department of Anesthesiology, The Ohio State University College of Medicine, 410 W 10th Ave, Columbus, OH 43210 USA; Department of Anesthesiology and Pain Medicine, Nationwide Children’s Hospital, The Ohio State University College of Medicine, 700 Children’s Drive, Columbus, OH 43205 USA; Department of Pathology and Anatomy, The Ohio State University College of Medicine, 410 W 10th Ave, Columbus, OH 43210 USA; Department of Pathology and Laboratory Medicine, Nationwide Children’s Hospital, 700 Children’s Drive, Columbus, OH 43205 USA; Department of Anaesthesia and Critical Care Medicine, The University of Toronto, 123 Edward Street, Toronto, ON M5G 1E2 Canada; Section of Anatomic Pathology, Department of Biomedical Sciences, Cornell University College of Veterinary Medicine, T5-006A Veterinary Research Tower, Tower Rd., Ithaca, NY 14853 USA

**Keywords:** Piglets, Neurotoxicity, Anesthesia, Neuroinflammation, Neurocognitive outcome, Neurodevelopment, Isoflurane, Hippocampus

## Abstract

**Background:**

Anesthesia-induced neurotoxicity research in the developing brain must rely upon an unimpeachable animal model and a standardized treatment approach. In this manner, identification of mechanisms of action may be undertaken. The goal of this study was to develop a novel, clinically relevant, translational way to use a piglet model to investigate anesthesia effects on the developing brain.

**Methods:**

29 newborn piglets were assigned to either: (1) control (no intervention, n = 10); (2) lipopolysaccharide (LPS; positive inflammatory control, n = 9); or (3) isoflurane anesthesia (n = 10). Positive inflammatory control animals were given 100 mcg/kg LPS from *Escherichia coli* intraperitoneally (IP) on the same day as those receiving isoflurane. Isoflurane was administered for 3 h while care was taken to ensure human perioperative conditions. To establish a clinical scenario, each animal was intubated and monitored with pulse oximetry, invasive and non-invasive blood pressure, electrocardiogram, temperature, end-tidal CO_2_, anesthetic concentration, and iSTAT blood analysis. All animals were sacrificed after 48 h using transcardiac perfusion of ice-cold, heparinized phosphate buffered saline (PBS) followed by 4 % paraformaldehyde (PFA). Brains were collected and histopathological analysis focused on the entorhinal cortex looking for degenerative changes due to its critical role in learning and memory. Reliable identification of entorhinal cortex was achieved by using colored ink on the surface of the brains, which was then cross-referenced with microscopic anatomy. Hematoxylin & eosin-stained high-power fields was used to quantify cells. ImageJ™ (National Institutes of Health, Bethesda, MD, USA) was used to count absolute number of progenitor glial cells (PGC) and number of PGCs per cluster. Immunohistochemistry was also utilized to ensure positive identification of cellular structures.

**Results:**

Histopathological sections of 28 brains were analyzed. One animal in the LPS group died shortly after administration, presumably from inadvertent intravascular injection. There was an acute basal ganglia ischemic infarct in one isoflurane-treated animal. A large number of small, round nucleated cells were seen throughout layer II of the entorhinal cortex in all animals. These cells were identified as PGCs using immunohistochemistry and light microscopy. Although there was no difference in the absolute number of PGCs between the groups, animals given isoflurane or LPS demonstrated a significant increase in cells forming ‘clusters’ in the entorhinal cortex. An apparent change in the pattern of doublecortin labeling also suggests changes in neuronal precursors and undifferentiated neurons.

**Conclusions:**

This study represents the first novel use of a clinically relevant neonatal piglet model to study anesthesia effects on the developing brain. LPS induces neuroinflammation, and this is a potential mechanism for LPS and perhaps isoflurane in causing a change in progenitor cell distribution. We postulate that the isoflurane-induced change in glial progenitor cell distribution could have important implications for cell differentiation, maturation and neural circuit behavior in the rapidly developing brain.

## Background

Each year, millions of children receive general anesthesia around the world. Isoflurane, a GABA type A (GABA_A_) receptor agonist, is an inhaled anesthetic commonly used in clinical pediatric anesthesia practice. Anesthesia is traditionally assumed to be safe as long as severe hypotension and hypoxia are avoided. Interfering with the balance of excitatory and inhibitory neurotransmitters in the developing brain may induce neuroinflammation and alter the formation of normal synaptic connection and dendritic arborisation. These changes could potentially lead to excessive loss of cells at critical times during brain development, and subsequently cause significant cognitive dysfunction. Recent reports of neurotoxicity-induced with isoflurane and other anesthetic agents in pediatric patients have triggered significant concerns about their safety, particularly in infants and young children [1].

Contemporary pediatric anesthesia is confronted with an urgent need to elucidate the potential neurotoxic effects of commonly used anesthetics on the brain in development. A recent Letter to the Editor published in *The New England Journal of Medicine* has generated significant concerns about anesthesia-induced neurotoxicity in children and reinforced the urgent need to identify the true meaning of these allegations [2].

It has become imperative for the pediatric anesthesia community to find proper anesthesia regimens in order to ensure safe anesthesia care for infants and children undergoing necessary surgical procedures and general anesthesia.

### Rationale for the piglet model

The need for a readily available, reproducible and translational animal model that is as closely applicable to human neonates as possible, allowing studies of the effect of anesthesia on the brain in development, is mandatory. Rodent and non-human primate models, used in most prior studies, are not ideal animal models to investigate anesthesia neuroinflammation/neurotoxicity for a number of reasons. Rodents are excellent models to investigate neuropathological mechanisms [3], but translating findings to humans is always a considerable challenge. While non-human primates may be excellent for the study of anesthesia-induced neurotoxicity, only a few pediatric anesthesia toxicity studies have been done in this model [4, 5]. Further, the use of non-human primates has several limitations that impede its broad implementation, such as cost and difficulty of handling.

In recent years, the piglet has emerged as a well-recognized alternative to larger animal models for neuroscience research. They have been used to study myriad pediatric diseases, including neuroinflammatory conditions (infection, stroke, traumatic brain injury, epilepsy) [6–9]. This is, perhaps, a result of the level of homology between human and porcine central nervous systems. Interspecies similitude begins with embryologic development. The timeline of neurodevelopment, though shorter than that of a human, is strikingly analogous to human brain development. There are parallels in neural tube closure, reelin expression, sex-specific development, gliogenesis, and neurotransmitter makeup [10–13].

Post-natally, there are numerous striking neuroanatomical and neurophysiological similarities when one compares the human brain with the piglet brain. Piglets share more CNS similarities with humans than any other mammal. For instance, humans and pigs are the only known mammalian species with a brain “growth spurt” that traverses the perinatal period, with the period of rapid development extending from the prenatal to the postnatal period [14]. Additionally, there are many gross anatomical features shared between pigs and humans, including character and distribution of brain gyri, gray matter, and white matter [15, 16].

The relevance of an animal model must be understood in the context of human pathology, particularly in relation to the brain maturity and pathobiology of the human infant. In the case of non-human primates, very few genes for neurotransmitter receptors have been cloned, meaning that crucial receptor-ligand affinities, allosteric modulators, post-translational modifications, alternative splicing variants, and receptor subunit compositions, all relevant to anesthetic-mediated neural injury, are unknown for this species.

On the other hand, porcine GABA_A_ and NMDA receptors have been cloned [17]. In contrast to newborn rodents, the percentage of adult brain weight at birth in piglets is much closer to that of humans [14]. The brain of the pig more closely resembles the human cerebrum as it is gyrencephalic whereas the rodent brain is lissencephalic. Neonatal swine cranial geometries and cortical and basal ganglia topology are similar to human infants. Important similarities between swine and humans have also been demonstrated in the hippocampus, basal ganglia, and brainstem, which will certainly be areas of interest for our study [18]. The pig and human brain are similar in growth patterns.

Brain development in the piglet is more analogous to that of full-term gestation human newborns than to non-human primates with prenatal brain growth spurts, or rodents with postnatal growth spurts [14]. The multilayer cortical sub-plate of the developing pig brain and the expression pattern of reelin, a glycoprotein that influences neuronal migration, are strongly analogous to the developing human brain [10, 19]. Grey and white matter distributions and patterns are similar in human neonates and piglets, and the maturation of the postnatal pig brain is comparable to humans with respect to myelination and electrical activity [18, 20]. Another important consideration is the time difference in brain development between piglets and human infants. Ten to 14-day old piglets show the rapid brain growth, neuronal maturation, and dendritic arborisations/connections comparable to a 2-month-old human infant [18]. Perhaps most importantly, an impressive homology exists between the genomes of humans and pigs, with many genes being conserved across both species [21].

There is a clear indication for a more suitable pre-clinical model to study anesthesia-induced neurotoxicity in the neonatal brain. Anesthesia-induced change in brain cell architecture provides proof-of-concept evidence for the suitability of the piglet model in neurotoxicity studies.

The need for a “positive control” for this study was evident, as little is known about the appearance or manifestation of neuroinflammation in the pig. As inflammation is one potential mechanism underlying toxicity seen with anesthetics, the LPS group was used to test whether an acute inflammatory response can induce significant changes in cytoarchitecture of the piglet brain and represent a benchmark for comparison for animals receiving isoflurane.

LPS are found in the outer membrane of gram-negative bacteria. These molecules are known to be strong immunogens in animals, and are capable of inducing a robust inflammatory response. The LPS used in this experiment is an endotoxin from *Escherichia coli* O127:B8 (Sigma-Aldritch, St. Louis, Missouri, USA), which has been used successfully to induce inflammation [22]. The systemic acute phase response in piglets is manifested by fever, lethargy, tachypnea, and diarrhea. It is clear that peripheral inflammatory stimuli lead to neuroinflammation, as evidenced by microglial activation and elevation in CNS cytokines [23].

## Methods

### Animals and animal handling

Healthy, domestic piglets (*Sus scrofa)* were used. Animals were 10–14 days old and 2–4 kg depending on the farrowing dates of sows and were obtained from a farm approved by The Ohio State University Institutional Animal Care and Use Committee (IACUC). All animal experimentations were performed in accordance with IACUC policy. Animals arrived in the vivarium 24 h before experimentation to allow them to acclimate to the environment. They were kept in temperature-controlled cages and given a commercial piglet milk replacer (Liqui-Wean, Milk Specialties, Eden Prairie, MN, USA) ad libitum. Routine animal care was provided by trained veterinary technicians and supervised by licensed veterinarians. The animals were randomly divided into three study groups: (1) control (no intervention, n = 10); (2) lipopolysaccharide (LPS; positive inflammatory control, n = 9); or (3) isoflurane anesthesia (n = 10).

### Control animals

**Fig. 1:**
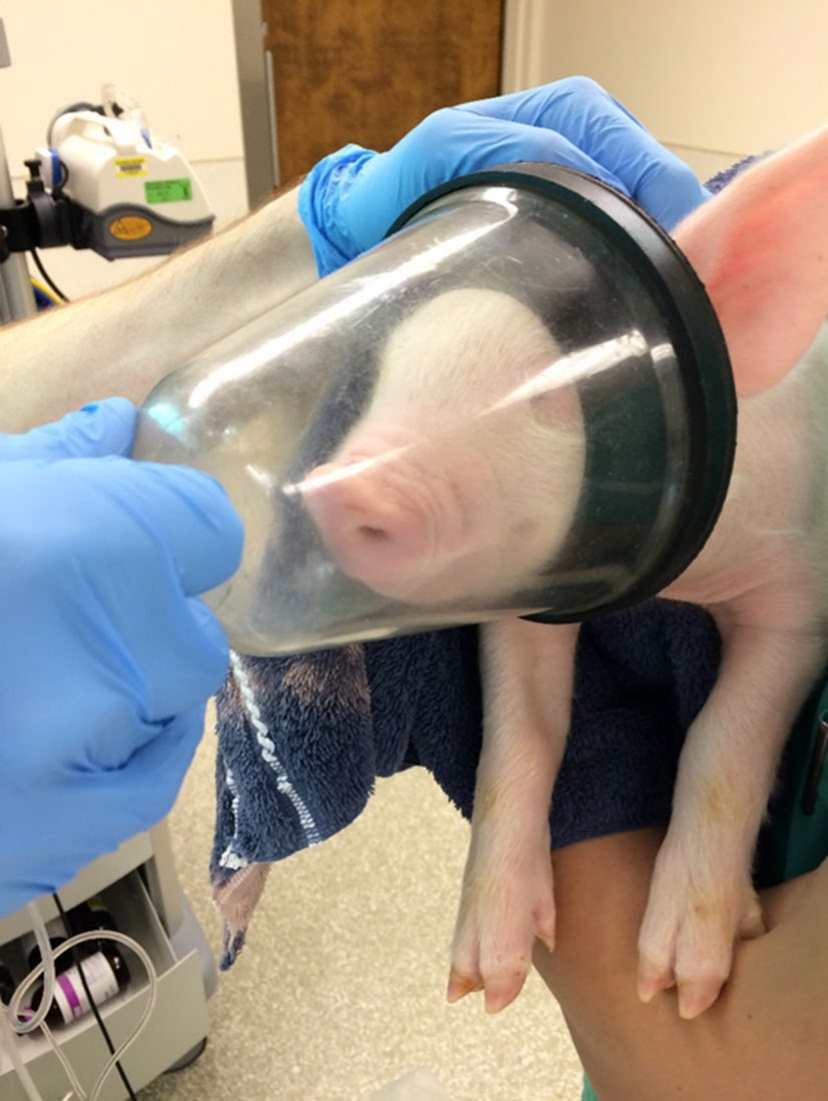
One piglet being administered an inhalational induction of anesthesia with 8 % sevoflurane in 100 % oxygen via face cone mask

Control animals received no experimental intervention of any kind. After 48 h, piglets were anesthetized briefly with 8 % sevoflurane in 100 % oxygen via face cone mask for sacrifice (Fig. 1).^1^ Sevoflurane was used for induction in order to mimic human procedures as closely as possible. This procedure was followed with all experimental groups. Adequate depth of anesthesia was confirmed by lack of dewclaw-pinch reflex. Transcardiac perfusion was performed using a commercially available peristaltic pump (Masterflex L/S, Cole-Parmer, Court Vernon Hills, IL, USA). The perfusate consists of cold (4 °C) phosphate-buffered saline (PBS) containing heparin (5 U/mL). The piglet was initially perfused with this solution at 300 mL/min for 5 min (or until the solution ran clear). Once the PBS perfusion was complete, a craniectomy was performed to remove one hemisphere of the brain for fresh tissue analysis. During this procedure, the circulation of ice-cold PBS was continued at a rate of 50 mL/h to ensure that the brain remained cold. Caution was used when removing the hemisphere to prevent disruption of the remaining hemisphere’s blood supply. The hemisphere was flash-frozen and stored at −80 °C for later analysis. After the hemisphere was removed, the perfusate was changed to 4 % paraformaldehyde (PFA). The PFA perfusion was continued at 300 mL/min for at least 5 min. Tissue fixation continues at least overnight in 4 % PFA at 4 °C. After 24 h, the fixed brain was moved to a solution of PBS containing 0.1 % sodium azide. Tissue was stored at 4 °C for up to 1 month.

### Inflammatory control animals

LPS was administered intraperitoneally at a dose of 100 mcg/kg. Once the LPS was administered, cutaneous temperature was continuously monitored using a liquid crystal strip thermometer affixed to the skin. Following administration of the LPS, animals were observed at least once an hour for the first 6 h by experienced staff. It is also important to provide an area that allows the piglet to remove itself from the range of the warming lamp. The most common manifestations of LPS administration in piglets were elevated body temperature (up to 105 °C), tachypnea, and anorexia, though vomiting and diarrhea can also be seen. No narcotics or anti-inflammatory medications were given. After receiving LPS, the piglets were allowed to convalesce for 48 h, after which piglets were anesthetized and tissue collected in the same manner as above-mentioned.

### Isoflurane anesthesia

A primary goal of this study was to develop a standardized approach to anesthetic administration in a complex animal model, with the aim of eliminating the confounding effects of hypoxia, hypercarbia, electrolyte abnormality, or other physiologic perturbation. A classic clinical anesthesia workstation (Integra SL Anesthesia Workstation, DRE Medical Equipment, Louisville, KY, USA) equipped with a pediatric ventilator and monitoring devices used to perform anesthesia in humans was considered critical in this study. Prior studies have used anesthetic chambers to induce anesthesia, with little or no monitoring equipment. This could lead to unrecognized physiological instability that could confound results. Care was taken to ensure rigorous attention to maintaining perioperative conditions identical to human anesthesia. After the 24 h acclimation period, piglets were anesthetized with 8 % sevoflurane in 100 % O_2_ via face cone mask (Fig. 1). This reproduces the technique used to induce anesthesia in infants and children. In addition, the use of sevoflurane leads to a faster induction of anesthesia in animals and a lower risk of laryngospasm.

Pulse oximetry, non-invasive blood pressure, and electrocardiography were continuously monitored during the induction period and at all times during the study procedure. After induction, sevoflurane was discontinued and isoflurane was titrated to a concentration that allows adequate depth of anesthesia permitting sustained spontaneous respiration (≈2–3 % end tidal concentration). Isoflurane was used for maintenance anesthesia because it is still largely used worldwide due to its favorable safety profile and low cost. A 24-gauge peripheral intravenous catheter was placed in the marginal ear vein to infuse 5 % dextrose in Ringer’s lactate at maintenance rate, i.e., 4 times the piglet’s weight in mL/h, and to administer cefazolin, 25 mg/kg, pre-incision to prevent surgical site infection.

Tracheal intubation was performed and chest ventilation confirmed. The femoral artery was catheterized to ensure continuous blood pressure measurement and facilitate blood sampling for the assessment of acid–base status, blood gases, and electrolytes hourly throughout the procedure using the iSTAT blood analysis system. (Abbott Point of Care, Princeton, NJ, USA). Anesthesia was maintained for 3 h. Normal acid–base and electrolyte values along with recommended actions for correction are summarized in Table 1. Vital sign perturbations such as hypotension, arrhythmia, hypo-/hyperthermia, and hypoxia were corrected if necessary to ensure hemodynamic stability at all times (Table 2).

**Table 1:** Normal arterial blood gases and serum electrolytes in piglets

Parameter	Normal range	Correction
pH	7.35–7.45	Acidosis/alkalosis is usually respiratory; correct with ventilator settings
pCO_2_ (mmHg)	35–45	Hyper/hypocarbia usually due to ventilator support, correct with ventilator settings
pO_2_ (mmHg)	200–220 (50 % FiO_2_)	Hyperoxia should be avoided due to possibility of oxygen free radicals. Hypoxia may be corrected by increasing pressure support or positive and expiratory pressure, or temporarily increasing FiO_2_
HCO_3_ (mmol/L)	22–33	Perturbations are typically respiratory in nature. Correct ventilator settings accordingly
Sodium (mmol/L)	129–143	Increase or decrease isotonic fluid infusion rate
Potassium (mmol/L)	3.9–4.1	Hyper/hypokalemia are rarely seen intraoperatively, mild perturbations do not need correction
Ionized calcium (mmol/L)	1.1–1.6	For hypocalcemia, consider calcium gluconate 30 mg/kg/dose titrated to laboratory parameters
Glucose (mg/dL)	100–200	Increase or decrease dextrose containing, isotonic fluid infusion rate
Hemoglobin (g/dL)	7–10	In the absence of acute blood loss during surgery, anemia is usually due to a dilutional effect from intravenous fluid administration. No therapy is required

**Table 2:** Normal ranges for vital signs in neonatal piglets

Parameter	Normal range	Suggested treatment of abnormalities
Systolic blood pressure (mmHg)	65–95	Intraoperative hypotension should be treated with an isotonic fluid bolus, 10–20 mL/kg titrated to effect. Hypertension likely represents inadequate depth of anesthesia, and anesthetic can be deepened with buprenorphine or other narcotic without confounding experimental results
Diastolic blood pressure (mmHg)	35–55	
Heart rate (beats per minute)	120–200	Bradycardia is rare in the absence of hypoxia. Tachycardia likely represents inadequate depth of anesthesia, and anesthetic can be deepened with buprenorphine or other narcotic without confounding experimental results
Oxygen saturation (%)	90–100	Most piglets will require pressure support if respiration is spontaneous under anesthesia. Increasing pressure support or positive-end expiratory pressure often corrects hypoxia in anesthetized piglets
Respiratory rate (breaths per minute )	30–60	Postoperatively, piglets with tachypnea often need additional pain medication. In LPS-treated piglets, tachypnea is commonly seen with the acute phase response and requires no treatment
End-tidal carbon dioxide (mmHg)	35–45	Titrate ventilator support as indicated
Core tempature (°C)	101–103	Hypothermia can be avoided by housing piglets in temperature-controlled cages and by maintaining active warming during surgery. Hyperthermia may be sign of malignant hyperthermia and should be taken seriously. Fever is common in LPS treated animals, and requires no treatment

At the conclusion of surgery, the femoral artery catheter was removed and the wound was infiltrated with 0.25 % bupivacaine, 1 mL/kg of with 1:200,000 epinephrine to control pain and to simulate human management. Anesthesia was discontinued and the piglet allowed to awaken, at which time the trachea was extubated. Buprenorphine 0.05 mg/kg was administered subcutaneously every 3 h as needed to ensure adequate pain control. When appropriate, the piglet was returned to its home cage, actively warmed with a warming light.

Animals recovered for 48 h. During this time, they were monitored every hour for the first 6 h after surgery and every 4 h thereafter. Monitoring includes general observation, temperature, inspection of the operative site, and measurement of respiratory rate. If the animal was awake and behaving normally, vital signs such as blood pressure were not measured due to the difficulty of obtaining non-invasive blood pressure on awake piglets.

### Histopathological analysis

**Fig. 2:**
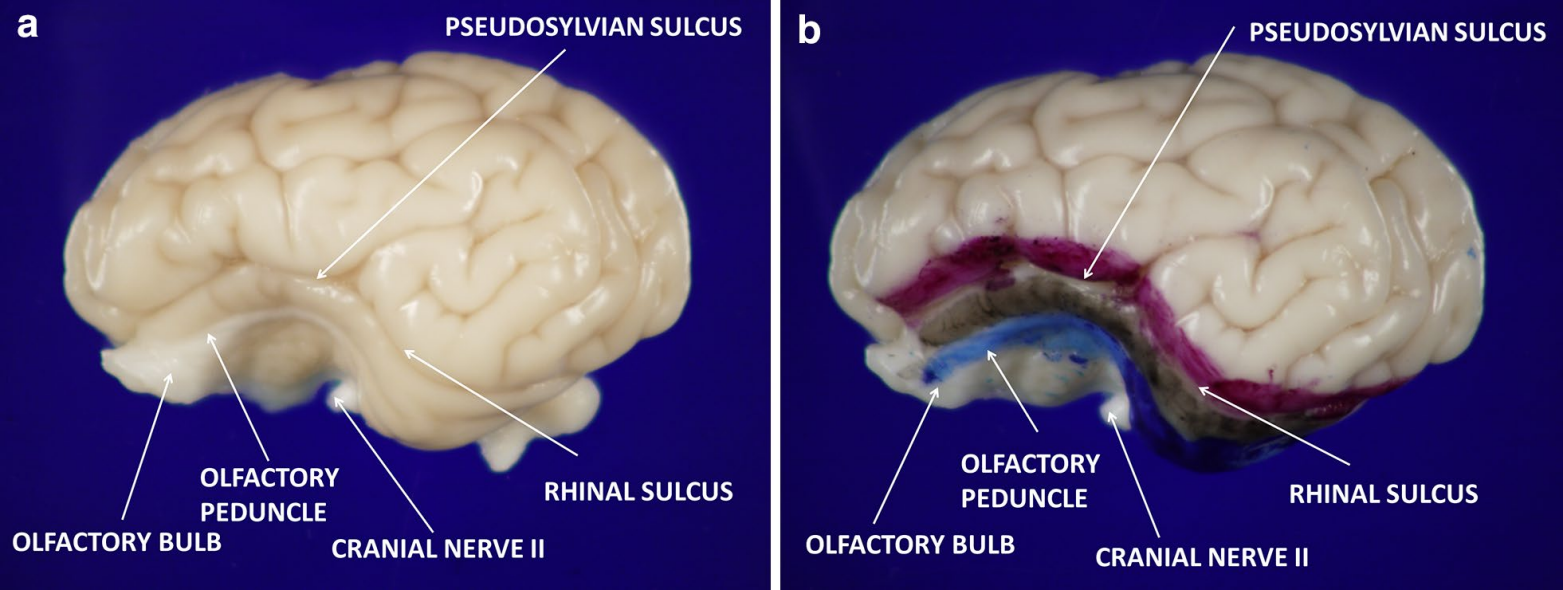
The gyrencephalic brain of the neonatal piglet. Panel **a** shows the left hemisphere without surface dye. Panel **b** illustrates surface dyes used to identify anatomical structures and guide microscopic evaluation. Structures are labeled based on conclusions drawn from cross-referencing dyed surface anatomy with known microscopic features of the entorhinal cortex

**Fig. 3:**
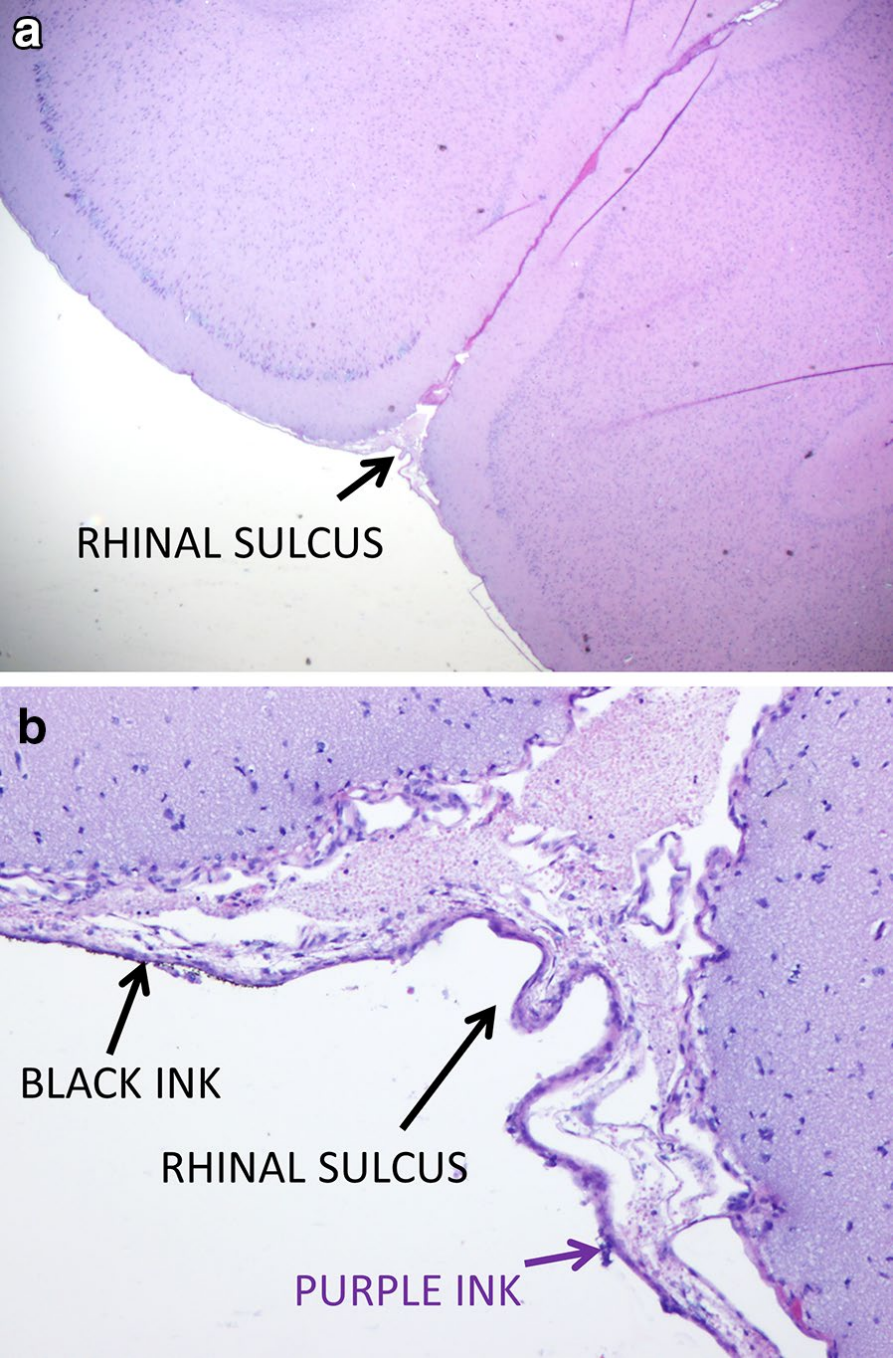
Microscopic anatomical brain tissue identification. Using dyed brain surface landmarks, the rhinal sulcus can be located at the junction of the *blue* and *black* ink (panels **a** and **b**). This information is used to assist in the identification of the entorhinal cortex, our primary area of interest

Fixed brains were sectioned by the same experienced neuropathologist using a standardized method to ensure that the same region of brain was studied for each animal. 10 µm sections were mounted and stained with hematoxylin and eosin. For each animal, six high-resolution, non-overlapping microscopic photos of the entorhinal cortex were taken at high-power magnification. Tissue dyes were used to mark the surface of the brain for microscopic correlation (Fig. 2). Dyes applied to the surface of the brain were visible on microscopic examination, and were used to identify microscopic features, such as our primary area of interest, the entorhinal cortex (Fig. 3). ImageJ™ software (National Institutes of Health, Bethesda, MD, USA) was used to count absolute number of PGCs and number of PGCs per cluster. A “cell cluster” was defined as a collection of more than 3 cells in direct contact with one another. An experienced clinical neuropathologist reviewed all sections and slides from control, LPS and isoflurane groups.

### Immunohistochemistry and quantification of doublecortin-positive cells

Fixed brains were sectioned and mounted using an identical method to that described above. Tissue was stained using anti-doublecortin antibody (Cell Signaling Technology, Danvers, MA, USA) and counterstained with hematoxylin and eosin.

### Statistical analysis

Demographic and parametric data are reported as mean ± SD. Analyses between groups were performed using an ANOVA and post hoc test for multiple comparisons. A Dunnett’s test was used to compare data with the control group whereas a Student–Newman–Keuls was used for between group comparisons. To ensure normal distribution and parametric nature of the data, the number of animals studied was determined by calculation of statistical power for a Type I and II errors of 0.05 and 0.8, respectively. A P < 0.05 was considered statistically significant.

## Results

Twenty-nine piglets were studied. All study procedures were well tolerated by animals with the exception of one piglet, which died within 10 min after injection of LPS. Because of the sudden and precipitous nature of its demise, it is believed that the piglet died as a result of inadvertent intravascular injection of LPS. All piglets studied were male. There was no significant difference between the groups with respect to age or weight (Table 3).

**Table 3:** Demographic characteristics of experimental groups

Group	Average age (days, ±SD)	Average weight (kg, ±SD)
Control (n = 10)	11.7 ± 3.06	3.05 ± 0.83
Isoflurane (n = 10)	11.6 ± 2.14	3.56 ± 0.83
LPS (n = 8)	11.6 ± 3.02	3.50 ± 0.76
*P* value	0.9967	0.2646

### Identification of small, nucleated cells

**Fig. 4:**
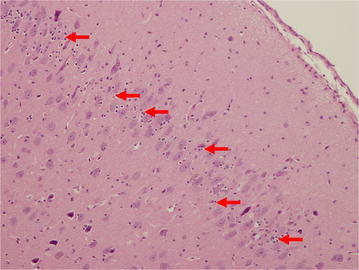
Entorhinal Cortex. Microscopic demonstration of brain tissue in a control animal at a magnification of ×100. The *red arrows* indicate the normal distribution of progenitor glial cells

**Fig. 5:**
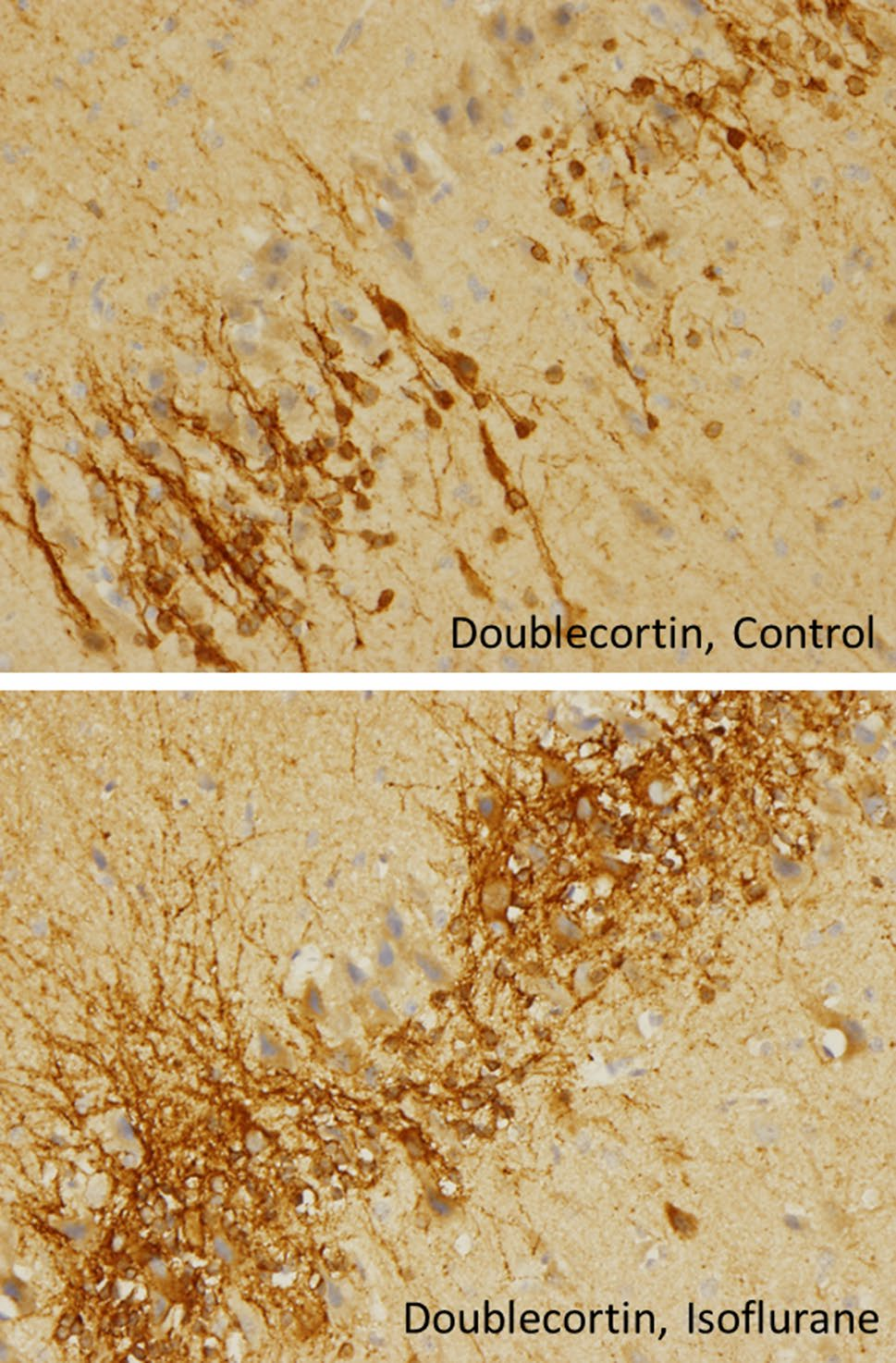
Doublecortin staining of immature neurons in entorhinal cortex. A significant increase in doublecortin positive cells is seen in isoflurane-treated animals when compared with control

10 brains were initially examined macroscopically in order to positively identify the neuroanatomical features to be studied microscopically. Microscopic brain tissue evaluation was performed [CP, AM]. Small, nucleated cells were commonly noted in control animals in and around bands of mature pyramidal neurons (Fig. 4). These cells are seen conspicuously clustered in the entorhinal cortex, neocortex, hippocampus, and periventricular zones. They are likely to be PGCs, a normal finding in immature mammals that may differentiate to become microglia, astrocytes, mature neurons, or oligodendrocytes. Identification of these cells was confirmed by immunohistochemistry for doublecortin, a microtubule-associated protein expressed by neuronal precursors and immature neurons. It was hypothesized that if the small, nucleated cells seen in the entorhinal cortex were PGCs, some, but not all, cells would stain positively for doublecortin because they had likely begun to differentiate. Indeed, overall, staining also suggests a different pattern of distribution and apparent increase in doublecortin immunoreactive cells and processes (Fig. 5). Quantitative analysis was not attempted given the complex pattern of distribution of doublecortin staining.

### Quantification of total PGCs in entorhinal cortex

**Fig. 6:**
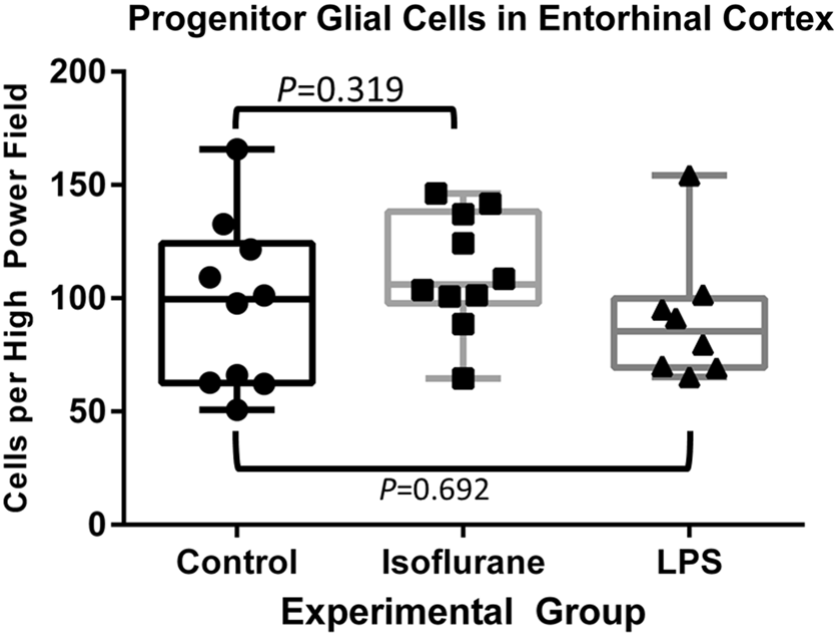
Quantification of PGCs in entorhinal cortex. Box- and -whisker plot displaying total PGCs in entorhinal cortex for all three groups. There was no difference in number of total PGCs when the isoflurane and LPS groups were compared to control

There was no difference in the absolute number of PGCs in entorhinal cortex in the isoflurane (*P* = 0.319) or LPS (*P* = 0.692) group when compared to controls (Fig. 6).

### Quantification of PGCs in clusters

**Fig. 7:**
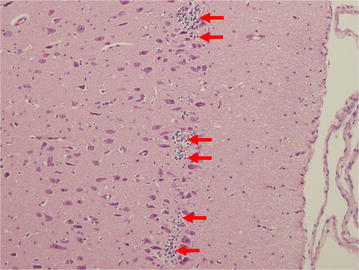
Clustering of PGCs. Microscopic view of layer II of the entorhinal cortex at ×100 magnification. The clusters of PGCs can be observed (*red arrows*)

**Fig. 8:**
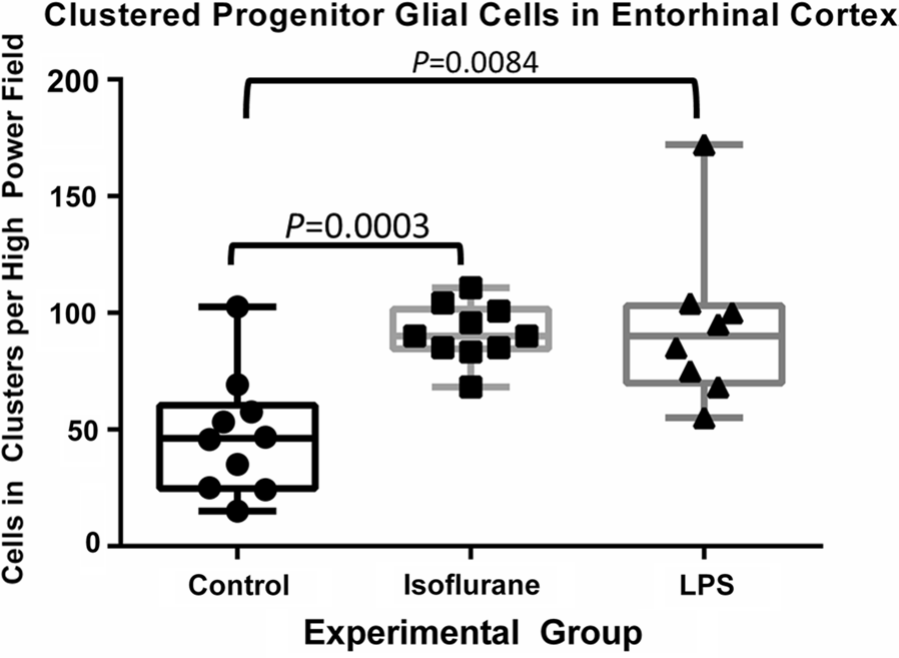
Quantification of PGCs in Clusters within the entorhinal cortex. There was a significant increase in the number of PGCs seen in clusters in both the isoflurane and LPS groups when compared to controls

PGCs were noted in clusters in all animals studied, but clustering was seen much more robustly in animals treated with isoflurane and LPS. A “cluster” of PGCs was defined as 3 or more PGCs in contact with one another (Fig. 7). Animals exposed to isoflurane exhibit a significantly different morphology and distribution of PGCs, including a quantitative increase number of cells seen in clusters when compared with controls (*P* = 0.0003). Interestingly, there was also a significant increase in PGC clustering seen in animals treated with LPS when compared with controls (*P* = 0.0084) (Fig. 8). These findings appear to be directly associated with the LPS or isoflurane exposure.

### Basal ganglia ischemic infarct in an isoflurane-treated piglet

**Fig. 9:**
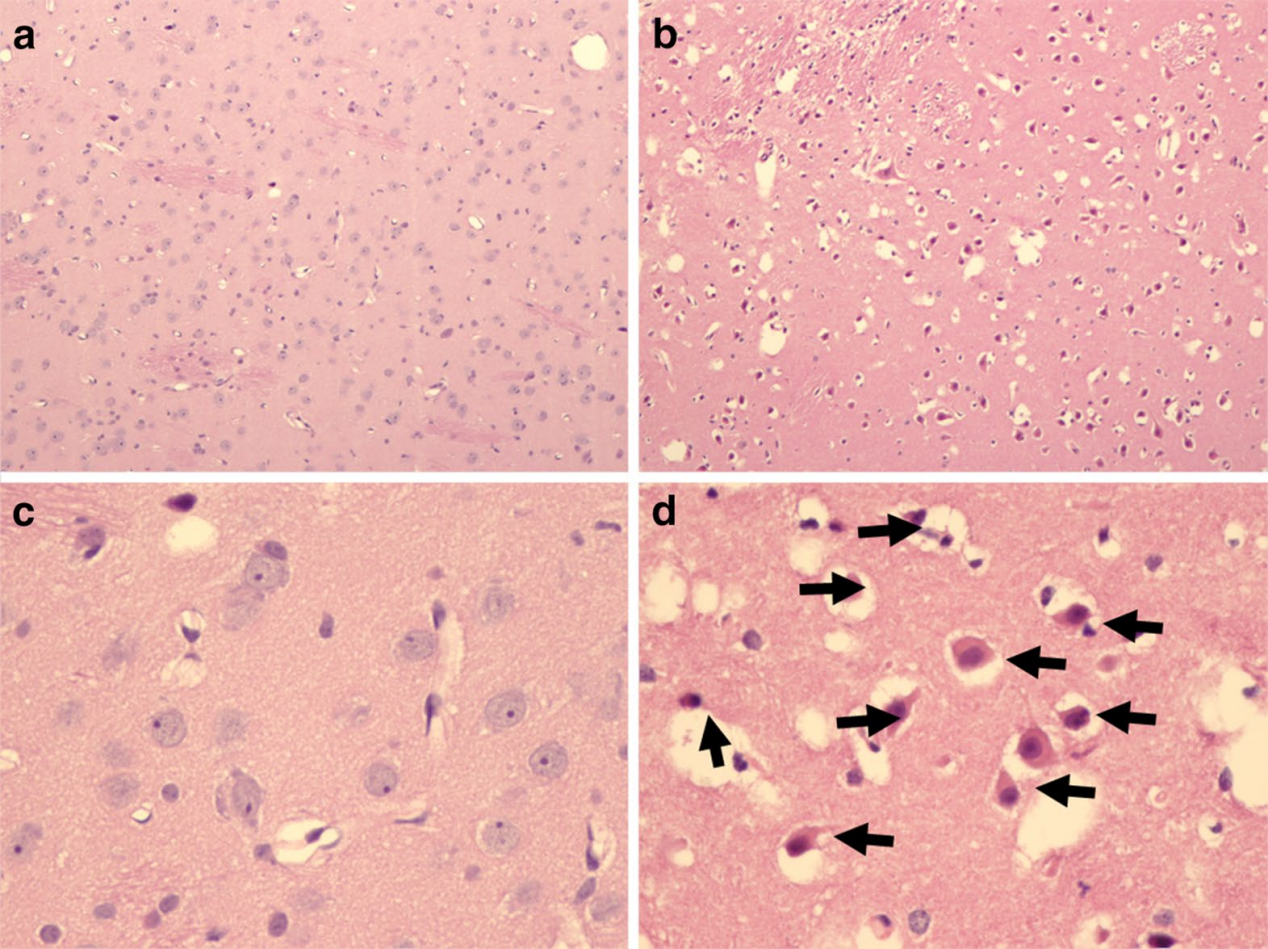
Basal ganglia infarct in an animal administered isoflurane. This composite of four panels shows a control animal (panels **a** and **b**) and an ischemic insult in an animal who was administered isoflurane (panels **c** and **d**). Panel **c** is characterized by the “shaggy” appearance of the brain tissue damage and hypereosiniphilic neurons (*black arrows*; panel **d**). Note the pericellular edema (*white halos*). This piglet suffered no periods of hypoxia or hypotension and electrolytes were normal at all times. It is critical to identify if this is an anesthetic effect

The second finding in the isoflurane-treated animals was an acute basal ganglia ischemic infarct in one animal. The infarct appeared to be 12–24 h old (Fig. 9) based on the lack of significant neuroinflammation and the acute neuronal necrosis.

## Discussion

This study represents the first novel use of a clinically relevant neonatal piglet model for the study of anesthesia effects on the developing brain. It reports, for the first time, changes in progenitor glial cell distribution in response to exposure to isoflurane anesthesia. We found that exposure to a clinically relevant administration of isoflurane, an inhalational anesthetic agent, induces a measurable increase in clustering of progenitor glial cells in an area of the brain that is critical for learning and memory. In addition, we report the concerning finding of an ischemic stroke in the basal ganglia of one animal exposed to isoflurane.

An important finding is that both isoflurane and LPS induction resulted in a significant increase in progenitor cells forming ‘clusters’ in the entorhinal cortex. These progenitor cells can differentiate into microglial cells, oligodendrocytes, astrocytes or neurons [24]. Therefore, a change in progenitor cell distribution implicates all these types of cells. LPS is known to induce neuroinflammation, activation of microglia and elevation of CNS cytokines [20, 21]. Therefore, one possible scenario is that isoflurane, like LPS, can induce changes in progenitor cell distribution in the piglet by activating a neuroinflammatory mechanism. Changes in the pattern of distribution of cells in the piglet brain may also involve neuronal precursors and undifferentiated neurons, identified by their doublecortin immunoreactivity. As noted earlier, 10 to 14 day old piglets show the rapid brain growth, neuronal maturation, and dendritic arborisations/connections that are comparable to a 2 month old human infant [16]. At this critical stage of rapid brain development, the observed alterations in the cellular organizational matrix of the entorhinal cortex strongly suggest that isoflurane (and LPS) are having a significant effect on the brain. Taken together, we postulate that the isoflurane-induced change in glial progenitor cell distribution could have important implications for cell differentiation, maturation and neural circuit behavior, as well as learning/memory in the rapidly developing brain. These novel questions can now be tested in a clinically relevant model.

Although the presence of progenitor glial cells in animals of this age is consistent with what is known about neurodevelopment in other, similar species, as well as humans, the changes in glial cell distribution is likely to be of importance. The consequence of alteration of the normal growth and distribution patterns of these cells is highly relevant to the question of anesthetic neurotoxicity. Though the neurocognitive effect of disrupting the PGC population is poorly understood, information gleaned from other models of disease underscore the importance of further investigating this finding. For example, the loss of glial cytoarchitecture in the gut has been shown to induce enterocolitis in mouse models of inflammatory bowel disease [25].

The entorhinal cortex was selected as an area of focus due to its importance in learning, spatial memory, and declarative memory. The entorhinal cortex serves as a communication hub between the cortex and the hippocampus, forming vital connections that allow for memory formation and processing. It receives afferent input from the sensory cortex, which in turn projects to the dentate gyrus, hippocampus, subiculum, and fimbria, successively. Thus, the entorhinal cortex is the “gateway” to the hippocampus.

Damage to this area could have significant and long-lasting effects on short- and long-term memory as evidenced by its critical role in the pathogenesis of Alzheimer’s disease. The implications of damage to this area of the brain are best underscored by the case of H.M., a patient who underwent medial temporal lobe resection for epilepsy in 1953. He lost virtually all of his entorhinal cortex, and as a result he suffered severe anterograde amnesia whereas his working and procedural memories remained intact [26].

The finding of an acute neuronal infarct in one piglet is extremely concerning, especially if it becomes a consistent finding in other piglets exposed to isoflurane anesthesia. Given the fact that brain infarcts in children are often “subclinical” and found incidentally when cerebral imaging is performed later for another reason, it will also be critical to identify the frequency of this occurrence. Kim et al. reported that 21 % of children had abnormalities, some of which were described as “unspecified white matter abnormality of unclear etiology” [27]. So-called “silent” strokes are shockingly common in children with sickle cell disease [28, 29]. It is not impossible that anesthetics, particularly when administered repetitively, may be causing areas of ischemic infarction that are never detected because they do not cause typical stigmata of stroke, such as weakness, facial droop, dysarthria, or gait disturbance. We are concerned that if subclinical infarcts are occurring during anesthesia, they may be partially responsible for the neurocognitive deficits that have been reported in children after multiple anesthetics [30]. It is vital to learn whether this is a common effect of anesthesia in animals with complex (gyrencephalic) brains in order to make anesthesia safer for infants and children.

We can now safely and effectively use this model to systematically test a number of different conditions and clinically used anesthesia regimens. This model represents the most clinically relevant animal model for neuroinflammation/neurotoxicity investigations. It reproduces with high fidelity the clinical scenario with respect to intubation, surgery, anesthesia, monitoring, and recovery infants and young children experience in the operating room and postoperatively, and its standardized approach carefully controls all physiologic variables that could confound results.

Although piglets are recognized as an ideal model for human infant neuroscience research due to their similarity in size, brain development, physiology, and pathophysiologic responses, our proof-of-concept study indicates that the piglet is a suitable model for anesthetic neurotoxicity screening [31, 32]. We strongly believe it is a remarkably well-adapted animal model, which is a major step toward understanding the potential for anesthesia-induced brain complications.

## Conclusions

The neonatal piglet represents a unique animal model to accurately investigate neurological anesthesia-related potential consequences considering its unparalleled similarities to the neonatal human brain. This clinically relevant, neurodevelopmental, neuroanatomical, and neurophysiological mammalian model provides striking similarities to human neonates. It is an accurate, highly reproducible, cost-effective, and appropriate to test a broad range of anesthetic regimens that are commonly used in contemporary pediatric anesthesia practice. One goal was to reliably induce neurotoxicity in the “inflammatory control animals” in a manner that will create a profile of neurocellular reactions that will be the benchmark for all future experimental investigations. Studies are underway to explore cell activation (i.e. microglial, astrocyte, neural), as well as global gene-expression profiles comparing control, LPS and isoflurane treatment groups to obtain a read out of the types of genes, including pro-inflammatory genes, channels, and pathways that may be sensitive to treatment.

The present study reports the effect of 3 h isoflurane anesthesia on progenitor glial cells of the entorhinal cortex, and provides a novel mechanism for investigation of the potential unwanted effects of prolonged anesthesia in the neonate piglet, that may potentially have similarities and implications for infant children receiving anesthesia. In previous studies, a connection was made between apoptosis and caspase-3 activation as a mechanism to explain anesthesia-induced neurotoxicity [5, 33, 34]. The latter mechanism remains to be tested in our model.

Further investigations will be needed to study later time points after anesthesia, multiple anesthetic regimens and their consequences on the brain in development. It is hoped that the resultant data will provide new insights into the extent to which anesthesia can induce neurotoxicity that would impact directly upon the safe practice of pediatric anesthesia. If so, scientific findings from this model could help identify the safest anesthetic regimens for infants and young children. It could also guide future neurological therapeutic interventions based on the identification of the mechanisms of action and molecular targets with the potential to ameliorate any anesthetic-induced neural injury.

## Footnotes

1 To avoid hypoxic/ischemic insults to the brain, a clinical anesthesia machine is used to ensure maintenance of adequate ventilation, oxygenation, and cardiac output until perfusion of cold PBS begins.
